# Establishing partnership with traditional birth attendants for improved maternal and newborn health: a review of factors influencing implementation

**DOI:** 10.1186/s12884-017-1534-y

**Published:** 2017-10-19

**Authors:** Tina Miller, Helen Smith

**Affiliations:** 1 Department of Social Sciences, Oxford Brookes University, Oxford, OX3 0BP UK; 2 Centre for Maternal and Newborn Health, Liverpool School of Tropical Medicine, L3 5QA, Liverpool, UK

**Keywords:** Traditional birth attendant (TBA), New roles, Partnership, Maternal health, Newborn health, Implementation

## Abstract

**Background:**

Recent World Health Organization recommendations recognize the important role Traditional Birth Attendants (TBAs) can play in supporting the health of women and newborns. This paper provides an analysis of key factors that affect the implementation of interventions to develop partnerships with TBAs to promote improved access to skilled care at birth.

**Methods:**

We conducted a secondary analysis of 20 papers identified through two systematic reviews that examined the effectiveness of interventions to find new roles for TBAs on maternal and newborn health outcomes, as well as papers identified through a systematic mapping of the maternal health literature. The Supporting the Use of Research Evidence framework (SURE) guided the thematic analysis to explore the perceptions of various stakeholders and implementation barriers and facilitators, as well as other contextual issues.

**Results:**

This analysis identified countries that have implemented interventions to support the transition from birth with a TBA to birth with a skilled birth attendant. Drawing on the experiences of these countries, the analysis highlights factors that are important to consider when designing and implementing such interventions. Barriers to implementation included resistance to change in more traditional communities, negative attitudes between TBAs and skilled attendants and TBAs concerns about the financial implications of assuming new roles. Facilitating factors included stakeholder involvement in devising and implementing interventions, knowledge sharing between TBAs and skilled birth attendants, and formalised roles and responsibilities and remuneration for TBAs.

**Conclusions:**

The implementation barriers identified in this analysis could, if not addressed, prevent or discourage TBAs from carrying out newly defined roles supporting women in pregnancy and childbirth and linking them to the formal health system. This paper also identifies the factors that seem critical to success, which new programmes could consider adopting from the outset. In most cases a multi-faceted approach is needed to prepare TBAs and others for new roles, including the training of TBAs to strengthen their knowledge and skills to enable them to be able to assume new roles, alongside the sensitization of healthcare providers, communities, women and their families. Further research is required to map the transition process and stakeholder experiences in more detailed ways and to provide longer-term monitoring of existing interventions.

## Background

Improving access to skilled care during pregnancy, childbirth, and postpartum remains a priority strategy for improving maternal health and is key to achieving one of the many indicators specified in sustainable development goal (SDG) 3, reducing the global maternal mortality ratio. In 2004 the World Health Organization (WHO), the International Confederation of Midwives (ICM) and the International Federation of Gynaecology and Obstetrics (FIGO) issued a joint statement defining ‘skilled birth attendants’ and their role. Although the statement did not include traditional birth attendants (TBAs), countries were encouraged to work with TBAs to define new roles for them and ensure cooperation between TBAs, skilled attendants, and staff in referral facilities [1]. Subsequent recommendations on optimising lay health worker roles [2], and WHO’s framework for working with individuals, families and communities [3] both identify a need to specify the responsibilities of TBAs for maternal and newborn health (MNH) in countries where they are an important source of childbirth care.

Specifying roles for TBAs acknowledges their cultural and social acceptability and the important role that they play in supporting the health of women and newborns and linking women, families and communities to the formal health system [3]. Strategies to increase use of Skilled Birth Attendants (SBAs) where TBAs are providers of childbirth care have generally involved linking TBAs with formal health services, increasing partnerships or teamwork with TBAs, or finding new roles. New roles for TBAs in the formal health system include encouraging and accompanying women to attend antenatal and postnatal care and have skilled care during birth, providing companionship to women during and after childbirth, as well as broader roles that they can play in community-level health education and community mobilization strategies to improve MNH. Strategies to increase partnerships with TBAs include developing collaborative relationships through opportunities for reciprocal sharing of traditional and professional knowledge between TBAs and SBAs, emphasising such collaboration in in-service midwifery training [4, 5], as well as including TBAs in community level networks and as links in referral chains for skilled care [6, 7].

Recent WHO guidance on health promotion interventions for maternal and newborn health endorses previous statements and recommends dialogue with TBAs, women, families, communities and service providers in order to define and agree alternative roles for TBAs in contexts where TBAs remain the main providers of care at birth [8]. The expert group reviewed the available evidence on the effect of establishing partnerships and new roles for TBAs on facility birth and birth with a SBA [8]. Overall, the evidence suggests improvements in use of SBAs, some increase in facility births, as well as some improvements in antenatal care (ANC) use and postpartum visits to women following the implementation of TBA interventions [8].

In the last 15 years countries have started to implement alternative roles and partnerships with TBAs, typically as part of complex and multifaceted interventions to increase skilled care which are difficult to evaluate. This makes it challenging to measure the contribution of TBA interventions, and to determine which strategies or which combination of strategies for TBA partnership are most effective [9, 10]. Nonetheless the recent WHO guidance recommends alternative roles for TBAs, and in this paper we turn attention towards understanding how best to implement these interventions. We summarise the available evidence and information on stakeholder perspectives and experiences in these interventions and the factors influencing implementation in different countries and contexts. This secondary analysis draws on studies from recent systematic reviews together with country experiences captured in descriptive accounts in order to synthesise: stakeholder experiences of partnerships and new roles for TBAs; the barriers and facilitators to implementation of partnerships and new roles; and how implementation factors relate to care seeking outcomes [8–10].

It is important to note the diversity in the country contexts in this paper and their stage of transition from TBA to skilled attendance. TBAs constitute a diverse group and may have had some basic training, for example when to refer a woman to a facility [4, 5] or be unskilled. They may also be described in different ways, for examples as ‘Traditional Midwives’ in Mexico and Nigeria [11, 12] and as ‘Dayas’ in Pakistan [13]. Detailed definitions of types of TBAs were not reported in the studies, but descriptions provided in the studies enabled a generic definition to be determined of a TBA as a non-professional, who has conducted home births, but lacks technical skills and training to manage obstetric complications. The TBA may have received training for the management of referrals [4–6].

## Methods

We conducted a secondary analysis of implementation factors identified in studies included in a systematic review of the effects of new roles and partnership with TBAs on MNH outcomes [8]. Our secondary analysis aimed to examine the included studies further to identify ideas about implementation that were not necessarily the central focus of the original research [14]. We considered the re-use of existing data an efficient way of generating new insights into implementing new roles for TBAs and extending the contexts in which the original qualitative research data could be interpreted [15]. The systematic review identified two existing reviews [9, 10] and included six studies identified through a mapping of maternal health literature published from 2000 to 2012 (focused on health system and community-based interventions for improving maternal health)[16]. For the analysis reported in this paper we include these six studies, which contained information about implementation, and they were quality assessed as part of the systematic review process. A further 14 sources were also identified, containing descriptions of implementation efforts to partner with or find new roles for TBAs; 13 of these from the inventory compiled in one of the above-mentioned systematic reviews [8–10], and another paper was identified during the more recent systematic mapping process reported above. These 14 sources contain useful information on countries’ experiences of implementing new roles or partnerships with TBAs; they mostly comprise review articles and grey literature without empirical data and so were not quality assessed.

The SURE (Supporting the Use of Research Evidence) framework was used to guide the extraction of information from papers and synthesis of factors influencing implementation presented in this article [17]. Dominant factors and recurrent themes were identified across the included studies and used to organise the data. These are reported below in relation to stakeholder perspectives and experiences as well as barriers and facilitators to implementation of new roles and partnership with TBAs.

## Results

A total of 20 papers comprising six studies identified through the systematic review process, and 14 additional sources, were included in this secondary analysis. The six research studies involved complex interventions, evaluated the use of different study designs and focused to varying degrees on improving MNH outcomes through interventions which included new roles for TBAs and TBA partnership [18]. Table 1 summarises the characteristics of these studies and the 14 additional sources. The countries represented in the six studies were: Bangladesh [6], Indonesia [4, 5], Myanmar [7], Peru [19] and Pakistan [13]. These studies report on various interventions to link TBAs with health services, increase partnerships with or find new roles for TBAs. Four studies evaluated community-based partnership interventions where midwives were trained and posted to work in poor, remote, or vulnerable communities and encouraged to work in collaboration with TBAs [4–7]. The other two studies evaluated new roles for TBAs within broader safe motherhood programmes; one culturally appropriate childbirth care model with TBAs and family members to encourage women to deliver in facilities [19] and the other multifaceted programme involved TBAs in raising awareness of services and reproductive health issues at community level [13]. The additional sources provided information on implementation of new roles or partnerships with TBAs in: East Africa (*n* = 3); West Africa (*n* = 1); South Asia (*n* = 1); South East Asia (*n* = 4); Central America (*n* = 3); South America (*n* = 1); and multiple countries (*n* = 1). The interventions described include: promoting partnerships between midwives, doctors, or health services and TBAs [11, 12, 20–24]; and creating new roles for TBAs including accompanying women to services [25, 26], supporting women during labour or childbirth [27, 28], and offering financial incentives to TBAs to refer women [12, 28].

**Table 1:** Characteristics of included studies and additional sources

Author	Study design	Setting	Description of intervention
Fauveau et al., 1991 [6]	Pre and post intervention study	BANGLADESH, rural Matlab	Nurse-midwives posted in outposts of programme area. The nurse-midwives’ duties included working with CHWs and TBAs to ensure that they were called during labour.
Frankenberg et al., 2009 [4]	Longitudinal panel survey	INDONESIA	A village midwife programme where midwives were trained and placed in poor communities far from health centres. Responsibilities included developing collaborative relationships with traditional village midwives.
Gabrysch et al., 2009 [19]	Pre and post comparative study	PERU, Ayacucho rural Santillana district	A culturally appropriate childbirth care model developed with Quechua communities and health professionals. Key features included a rope and bench for vertical delivery position, inclusion of family and TBAs, use of the Quechua language and health professionals that were respectful of culture.
Mullany et al., 2010 [7]	A pre and post intervention study (2-stage cluster-sampling surveys before and after programme implementation)	MYANMAR, Shan, Mon, Karen, and Karenni regions	A community-based project, collaboration to improve coverage of maternal health services in vulnerable communities. Strategy of a 3-tiered network of community-based providers: TBAs created links between community member and senior workers, HWs strengthened links between TBAs and lead health workers, and MHWs, who oversaw the work of TBAs and other HWs.
Purdin et al., 2009 [13]	Programme evaluation using health information system/retrospective pre-post analysis	PAKISTAN, Hangu district, Afghan refugee camp	Male involvement interventions relating to safe motherhood, services established, and training provided to different community actors including TBAs to raise awareness of services and reproductive health issues. Outreach and community input. Each refugee camp has a health committee with community representatives and health service staff that meet bi-monthly to discuss project activities and provide feedback to health workers on health services provided. Basic emergency obstetric care facilities, community-based education provided to key community representatives including CHWs, female health workers, women of reproductive age, men, health committee members (all men), teachers, religious leaders and private practitioners.
Ronsmans et al., 2001 [5]	A pre and post intervention study, including mixed methods	INDONESIA, three districts in South Kalimantan	A midwife placed in each village and at least one doctor with obstetric skills in each district. Financial access was facilitated for the poor. Village midwives were encouraged to work side by side with TBAs. TBAs supported to refer women with a complication and to work in collaboration with village midwives.
Additional sources			
Andemichael et al., 2009 [25]	Published article – rapid assessment	ERITREA	Improve access to services: addressing geographical barriers. New role for TBA: accompanying women to health services.
Braine, T. 2008 [11]	Published article - opinion	MEXICO	New role for TBA: Doctor + TBA partnership. Cultural adaptation of institutional childbirth.
Davis-Floyd, R. 2001 [30]	Published article – qualitative research	MEXICO	Improve access to services: addressing geographical barriers. New role for TBA: midwife + TBA partnership.
Fonseca-Becker et al., 2004 [27]	Published (online) report	GUATEMALA	New roles for TBA as a key component of newly established health committee. Recognises TBAs importance as a (culturally acceptable) bridge between pregnant women and the health system. TBAs incorporated into activities in health facilities as part of MNH. TBA giving emotional support and coaching during labour, passing instruments to SBA and acting as interpreter.
Koblinsky et al., 1999 [28]	Published article – review paper	MULTIPLE COUNTRIES	Improve access to services: addressing geographical and financial barriers. New role for TBA: support at childbirth with health workers.
Murigi, S.F. 2010 [26]	Newspaper article	UGANDA	TBAs prohibited for childbirth. New role for TBA: community advocacy and accompanying women to health services
Onuki, D. 2002 [29]	Published article - opinion	BOLIVIA	New role for TBA: paid to refer pregnant women to health services. Cultural adaptation of institutional childbirth.
Ministry of Health, Myanmar 2010	Personal communication	MYANMAR	Improve access to services: addressing geographical barriers. New roles for TBA: community advocacy; health services + TBA partnership.
Ministry of Health, Nigeria, 2010	Unpublished concept note	NIGERIA	New role for TBA: community advocacy, accompanying women to services, support for women during labour and childbirth, paid to refer or accompany women to health services, provide a link between women and families and health services.
Ministry of Health, Southern Sudan, 2009	Unpublished report	SOUTH SUDAN	New role for TBA: accompany women to health services, financial incentives to TBAs, midwife + TBA partnership.
Save the Children, 2008	Unpublished report	AFGHANISTAN	Human resources development and deployment Policies to increase access to services, quality of the services, community participation and midwife + TBA partnership
UNICEF Indonesia, 2010 [21]	Published (online) report	INDONESIA	New role for TBA: midwife + TBA partnership.
Weber, M. 2010	﻿Innovation ‘letter to a friend’ (‘surat dari sahabat’). Also described in reference [21]	INDONESIA	New role for TBA: midwife + TBA partnership. Community advocacy.
World Bank, 2010 [22]	Published (online) report	INDONESIA	Human resources development and deployment Improve access to services: addressing geographical and financial barriers. New role for TBA: midwife + TBA partnership.

### Stakeholder experiences of partnerships and new roles for TBAs

The stakeholders most often identified in the interventions are TBAs, community-based SBAs (trained midwives), facility-based SBAs, women service users, family members and local communities including men and religious leaders. The perspectives of each of these groups on partnership and new roles for TBAs are not detailed in depth in any of the included sources, but our analysis identified some insights into how these interventions are received and experienced.

#### TBA, community-based SBAs, and facility-based staff experiences

Interventions to develop collaborative partnerships between TBAs, SBAs and facility-based staff have involved relationship-building integrated into midwifery training in Indonesia [4], and integration of TBAs into a ‘referral chain’ with nurse midwives in Bangladesh [6]. TBA-facilitated sessions on traditional birth and medications and village midwives working ‘side-by-side’ with TBAs have been used to promote partnership in Peru [19] and Indonesia [4, 5] respectively, and in Sudan incentive schemes to motivate TBAs to work collaboratively have been introduced [24]. In Myanmar links with TBAs have been strengthened through a network of community-based provision in which maternal health workers have responsibility for overseeing the work of TBAs [7].

Reports suggest that TBA experiences of new partnership working with SBAs and facility-based staff are varied. In Guatemala, TBAs describe their increased confidence, for example when giving information to families and persuading husbands to support use of facility care [27]. But others in the same programme report suspicion of their new roles by some members of their communities and a lack of consideration for their personal needs when accompanying women to a hospital facility (e.g. food and accommodation) [27]. Further evidence from the Guatemala case study suggests that TBAs collaborate with the facility and accompany women as an ‘obligation’ to women who need help, and because it helps give the hospital a ‘good image’ with the population [27]. In Malaysia collaborative working between TBAs and SBAs was reported to have improved previously poor relations as TBAs had been gradually shifted to ‘a more exclusively family supportive role’ since 1973 [28].

There are also reports of facility staff having a low opinion of TBAs, and thereby making them feel unwelcome [27]. But improved relationships between TBAs and facility personnel were reported in Mexico and Peru [11, 19] following a broad participatory approach, and the design of a culturally appropriate model of care which included TBAs in Peru [19]. Integrating TBAs as birth companions (to give traditional massage and drinks) [11, 19] and as interpreters [19, 28] was accepted positively by facility staff and TBAs.

#### Women service users, family members and wider community experiences

Few papers report directly or in detail on women’s experiences of new roles or interventions to promote partnerships with TBAs. The need to include ‘key decision-makers’ from communities including men (who are often household decision-makers) and religious leaders in TBA partnership interventions was noted in some papers [5, 6, 13], but only scant reporting of their experiences was included in the analysed papers. Involving household stakeholders (women, mothers-in-laws and husbands) and TBAs in assessing reproductive health information materials was a strategy used in Indonesia to foster collaboration [5]. Community empowerment and an increase in men’s awareness of maternal and neonatal health was also reported following partnership working in Indonesia [21]. The need to establish ‘strong links with the community’ when implementing changes to models of care was reported more generally [28] and in particular the importance of community ‘consideration’, ‘consultation’, ‘mobilisation’ and ‘inclusion’ noted in relation to TBA partnership in Peru [19], Nigeria [12], Guatemala [27] Myanmar [7] and Indonesia [4, 5], even though detailed reporting of community stakeholder experiences was limited, with the exception of a case study conducted in Guatemala [27]. Some studies emphasised the value of ‘intercultural adaptation’ [29] and ‘culturally appropriate’ skilled care provision which retained TBAs as companions and interpreters [11, 19, 27, 30]. For example, in Guatemala, hospital workers were observed to show more patience, consideration, and respect to the women using their services, and for women, having access at the facility to an interpreter who spoke their language was ‘deemed extremely positive’ [27]. Trust between the hospital staff and local women was reported to have increased as a result of the changes to the organisation of care including recognising TBAs as a culturally acceptable bridge between women and the health system [28]. In Peru, family members being allowed to stay at the facility was also regarded positively by the families [19]. But in Myanmar concern was expressed by some family members about the financial costs (including loss of work time) and disruption to family life associated with childbirth at a facility and the need to leave the family home [23].

### Barriers and facilitators to implementation of new roles for TBAs

Implementation challenges exist at the individual, community, facility and wider health system levels. The factors identified in the papers broadly focus on the importance of sensitising key stakeholders in order to change traditional TBA practices and achieve partnership with health service personnel, shifting attitudes and strengthening working relationships between TBAs and other health workers, and finding ways to adequately reward and incentivise TBAs in their new roles. It was also recognised that it could take time for new ways of working to become accepted and for behaviours to change.

#### Changing traditional TBA roles and practices

The continued adherence to traditional practices and a ‘greater degree of traditionalism’ in some communities [4] and countries [6, 23] have been identified as barriers to change and reasons why TBA partnership and anticipated shifts to SBA have not occurred in ways, or at a pace, anticipated [4, 28]. A continued preference for TBAs and traditional birth practices was noted in Eritrea [25] and Myanmar [23] and cultural practices in some regions [11, 19, 20, 23, 27, 28, 30] have been recognised as a barrier to skilled care use as women and community members still trust and prefer to use TBAs. A lack of available or accessible skilled care in some remote communities is also a reason given for continued use of TBAs [21–23, 26].

In contrast, several countries have experienced gradual shifts in traditional practices and cultural beliefs towards acceptance of new roles for TBAs and greater trust in midwives for technical aspects of childbirth [21]. This has been achieved through sharing of knowledge between TBAs, community-based SBAs, and facility-based staff and through community involvement especially in relation to designing culturally-appropriate facility care in Peru [19], Guatemala [27], Mexico [11, 30] and Bolivia [29]. This has led to improved relationships between multiple stakeholders [19]. But changing traditional practices in some communities has been slow [4, 26, 28] and community involvement was widely identified as essential in changing behaviours towards skilled care at birth [11, 19, 27]. The inclusion of key decision makers, including men, in information campaigns to increase demand for skilled care at birth and raise awareness of new roles for TBAs was also noted in Pakistan [13] and in Indonesia [5]. In Peru using an inclusive, ‘participatory approach’ to identify local needs facilitated the introduction of culturally-appropriate skilled care, and ensured that service use was sustained [19]. Involving TBAs and community members in the assessment of information materials as a means to facilitate partnership and community support was reported in Indonesia, where local radio was also used as a community information tool [5].

#### Working relationships between TBAs and other health workers

As new roles for TBAs lead to closer working with SBAs and facility-based staff, rivalry and negative attitudes among the different cadres have been reported [5]. A review paper summarising different models of childbirth care highlights that in countries where home birth is predominant and the government attempts to link with TBAs, they are likely to view government staff as competitors for the same clients; government health workers on the other hand may have a low opinion of TBAs [28]. There are however examples in the included studies of how TBAs and other health workers have developed mutually respectful working relationships. For example, in Brazil TBAs were made to feel part of the formal health system by being granted access to a telephone for arranging transport to a facility and (in some cases) provision of a uniform, and this reportedly helped to overcome negative attitudes towards TBAs among skilled birth attendants [28]. In Myanmar integrating TBAs into a three-tiered network of care providers [7] and allocating them specific roles in providing antenatal care, conducting uncomplicated births, and creating links with upper-tier workers helped to promote good partnership working within the context of camps for internally displaced populations in the eastern border. The supervision of partnership working together with strong referral mechanisms was acknowledged more generally as a way to facilitate good working relations [28]. As part of the TBA-midwife partnership project in Indonesia, TBAs no longer help women give birth but their ‘social status’ has been retained helped by the formal acknowledgement of the TBA in the health system [21]. In many countries identifying new roles for TBAs (for example adapting facility care to include TBAs as birth companions) involved consultation with all stakeholders, which was found to facilitate better partnership working and helped to overcome rivalry between TBAs and skilled attendants [7, 11, 19, 27, 28, 30]. Many reports emphasised the importance of defining new roles for TBAs (for example, ANC, family health, postpartum care, breastfeeding and weaning, as advocates for MNH needs) and committing to these at the policy level [12]. The introduction of formal agreements (‘contracts’) between TBAs, SBAs and health services was reported to facilitate implementation in Brazil [28] and Indonesia [21].

#### Rewards and incentives for TBAs in new roles

As TBAs take up new roles and transition to partnership working they can experience an erosion of income and payment-in-kind previously received for conducting childbirth in the community. Performing new roles such as referring and accompanying women to facilities [25–27] and acting as a birth companion also involves new costs (e.g. transport and food) which some TBAs expressed concern about [27]. The need to ensure financial remuneration for TBAs was noted in several countries [4, 12, 28] and addressed through a formal signed agreement in Indonesia [21]. In Bolivia [29], Sudan [24], Indonesia [4] and Nigeria [12] payment for TBA referral or post-partum care [4] was introduced or recommended. Paid referral included encouraging TBAs to provide lists of pregnant women and referring women for skilled care in Indonesia [4], Peru [19] and Sudan [24]. In Indonesia, TBAs were encouraged to work collaboratively with midwives and report pregnancies to the health centre so that midwives could invite the woman for antenatal care by personal letter [21], and in Guatemala TBAs agreed to prepare lists of pregnant women for the health committee [27]. In Bolivia TBAs received payment for referral of pregnant women to a medical institution [29]. TBAs were also encouraged to refer women to Maternity Waiting Homes in Eritrea [25]. The introduction of performance based incentives for TBAs was recommended in Nigeria as a means to build capacity in their new social support role [12].

## Discussion

Our analysis has identified that in countries where TBAs have been encouraged to work in more collaborative ways with formal health systems, TBAs, community-based SBAs, and facility-based staff had mixed experiences of the interventions. As old practices change and new ways of working in partnership are introduced this can affect working relationships between TBAs and SBAs. For example, our analysis highlights rivalry and competition for the same clients and implies unequal power relations between TBAs and formal health workers who may have a low opinion of TBAs. Community suspicion of new roles, poor working relationships with SBAs, and not being made welcome at facilities are some of the challenges TBAs face in their new roles and working alongside skilled birth attendants. However, when programmes used broad participatory approaches to design new models of care which included TBAs, and where TBAs were given clearly defined roles (such as birth companions or interpreters for women during labour and birth) they were more readily accepted by facility staff and TBAs. The included studies contained little information about the experiences of women service users, families, and communities of alternative roles for TBAs. However, when programmes placed emphasis on culturally appropriate skilled care and included roles for TBAs, this was regarded positively by women and families who may previously have felt emotionally unsupported by SBAs [19, 27, 31]. In services that included TBAs as a bridge to women and communities trust between women and facility staff increased. Barriers and facilitators to changing traditional TBA practices and achieving partnership with SBAs and facilities were also identified in the literature [4, 6, 8–10, 23, 28]. These included strengthening working relationships and reducing suspicion between TBAs and other health workers, and adequately rewarding and incentivising TBAs in their new roles.

### What factors are critical to the success of TBA interventions?

To better understand the factors critical to the success of interventions to introduce new roles for TBAs, we considered the perspectives and implementation factors identified in this analysis in relation to the care seeking outcomes (birth with a skilled birth attendant or facility birth; ANC use (one to four visits) and postnatal visits for the woman) reported in the original systematic review. Table 2 lists the perspectives and implementation factors that made a difference in contexts where care seeking outcomes improved, together with key barriers to success. Of the six studies included in the original systematic review, three studies [4, 7, 13] reported improvements in number of ANC visits and coverage following the implementation of a TBA intervention and four of the six studies [5–7, 13] showed that postpartum visits to women increased over time following the implementation of a TBA intervention. Where TBA partnership interventions were reported to improve care-seeking outcomes, the interventions included community involvement, both to ensure culturally appropriate service provision, and community acceptance of new roles for TBAs. Within this dialogue, the involvement of men as key decision-makers in households was important to help change traditional care seeking behaviours. Bringing TBAs and skilled birth attendants together to work in partnership is best achieved where individual roles are clearly defined and supervised. Formal recognition of new roles for TBAs within health service systems also helps community acceptance of new responsibilities for example a broader, more family supportive role in the community. Formal recognition will also maintain the social status of the TBA.

**Table 2:** Implementation factors linked to systematic review outcomes

Studies from systematic review that report overall improvement in care seeking outcomes		Findings from synthesis of factors influencing implementation	
Author, year	Country, setting	Facilitating factors critical to successful outcomes	Barriers to successful outcomes
Fauveau et al., 1991 [6]	BANGLADESH, rural Matlab	Collaborative working between trained midwives, nurse-midwives, CHW’s and TBAs - Integration of TBAs into a ‘referral chain’ working with skilled personnel	- Continued adherence to traditionalism and traditional practices - Men as main decision-makers in the family may be unwilling to seek external assistance
Frankenberg et al., 2009 [4]	INDONESIA	- Collaborative relationships with TBAs identified as a duty of trained midwives posted in villages	- Greater degree of traditionalism in some villages where trained midwife posted
Gabrysch et al., 2009 [19]	PERU, Ayacucho rural Santillana district	- Adopting a participatory approach including TBAs and family members to design culturally appropriate childbirth services - TBAs accommodated as birth companions to help with pushing and giving drinks and to provide a culturally appropriate facility birth - TBAs involved in capacity building workshops with other health workers	- Providers continuing to ascribe high levels of home births as due to ‘cultural preference’ or ‘ignorance’ rather than offering culturally accessible services
Mullany et al., 2010 [7]	MYANMAR, Shan, Mon, Karen, and Karenni regions	- Integration of TBA into a 3 tiered network of community based providers - Training of HWs and TBAs to strengthen their working relationship - TBAs used to create links between community members and senior health workers - TBAs used to provide improved ANC - Supervision of TBA role a responsibility of MHWs	
Purdin et al., 2009 [13]	PAKISTAN, Hangu district, Afghan refugee camp	- TBAs provided with reproductive health (RH) training along with other community members - TBAs active in awareness raising, including RH	
Ronsmans et al., 2001 [5]	INDONESIA, three districts in South Kalimantan	- Village midwives encouraged to strengthen their relationships with TBAs - TBAs identified as one of several ‘key decision-makers’ - TBAs involved in accessing information, education and communication (IEC) materials for community use	

### Limitations

Owing to the comprehensive search strategies employed in the systematic reviews and systematic mapping exercise, we are confident we have located the available literature, published and unpublished, on implementation of TBA partnerships and new roles, albeit limited to publications written in English. There is a possibility we may have missed reports or evaluations from projects or programmes in South or Central America or West Africa. Our findings indicate important implementation considerations common across different contexts, but our understanding of how these factors change over time or how they interrelate to prevent or promote skilled care at birth is limited. None of the studies reporting evaluations of TBA interventions explicitly collected data on stakeholder preferences or attitudes towards the interventions, nor did they provide detailed descriptions of the TBA interventions such as how partnerships were built or strengthened and how new roles were identified and by whom. This could be because the TBA interventions were implemented as part of larger community or facility based programmes to increase skilled attendance and facility birth, and the evaluations focused on the programmes overall and not the contribution of the component parts. The additional and largely unpublished sources included in this analysis describe to some extent the nature of partnerships between SBAs, facilities and TBAs, but very little is revealed about how to nurture and sustain collaborative working, or the views of women service users, TBAs, and health workers about acceptability and appropriateness of the new roles and partnerships. Although there is an obvious value and efficiency in using already collected data to answer additional research questions, secondary analysis can pose a threat to fidelity in interpretation of findings beyond those presumed in the original research [ref Thorne 1998]. The themes identified in this paper reflect our critical analysis of data already interpreted and contextualised by authors of the primary studies. However we feel the threat is minimal as we did not set out to build theory or develop a higher order analysis, we simply extracted information on themes that primary authors had identified themselves.

### Research implications

Longer-term monitoring of TBA interventions, as new roles and partnership working become established, could help programme planners and policy makers identify what works and direct resources towards improvements or modifications. Better reporting of intervention characteristics including details of the type of TBA could help other programmes or countries decide what aspects to replicate or implement. There is also a need for more in-depth qualitative research and case studies, focusing on multi- stakeholder perspectives, to better understand what aspects of interventions (in different country settings and populations) facilitate acceptance of new roles for TBAs and lead to improvements in use of skilled childbirth care. Future evaluations could usefully explore and record the preferences articulated by those providing and receiving care in more detailed and rigorous ways. We would encourage publication of case studies from countries that have implemented policies to ban TBAs and the effect of these policies, as well as those countries with experience in changing the role of TBAs but which may not yet be documented in the literature, such as in Latin America. Future studies should include an estimation of costs, since exact costs were not reported in the literature we reviewed. We could infer from the different studies only limited information about costs incurred. These were dependent on the intervention type, including provision of payments to TBAs to take on new roles such as referring pregnant women, accompanying them to the facility, reimbursing transport costs or other costs associated with assuming a new role. It is also important to consider any additional cost burden to women and families, especially if there is an expectation that they should pay or compensate TBAs for helping them to access services for example. This is particularly relevant in countries where maternity care is not provided for free.

## Conclusion

Interventions to support countries to transition from birth with a TBA to birth with a SBA are important and timely in light of priority strategies to increase skilled care at birth and the human resource challenges in achieving this goal. The implementation barriers identified in this analysis could, if not addressed, prevent or discourage TBAs from carrying out newly defined roles supporting women in pregnancy and childbirth and linking them to the formal health system. This paper also identifies the factors that seem critical to success, which new programmes could consider adopting from the outset. In most cases a multi-faceted approach is needed to prepare TBAs and others for new roles, including the training of TBAs to strengthen their knowledge and skills to enable them to be able to assume new roles, alongside the sensitization of healthcare providers, communities, women and their families. The integration of other stakeholders to contribute to a support and monitoring system for the transition period is also recommended. As with many other interventions for improving maternal and newborn health, the availability and accessibility of skilled care and other health systems challenges remain important barriers; not least identifying a sustainable source of funding to compensate and incentivise TBAs in their new roles.
